# Deep learning for ECG Arrhythmia detection and classification: an overview of progress for period 2017–2023

**DOI:** 10.3389/fphys.2023.1246746

**Published:** 2023-09-15

**Authors:** Yaqoob Ansari, Omar Mourad, Khalid Qaraqe, Erchin Serpedin

**Affiliations:** ^1^ ECEN Program, Texas A&M University at Qatar, Doha, Qatar; ^2^ Weill Cornell Medicine-Qatar, Doha, Qatar; ^3^ ECEN Department, Texas A&M University, College Station, TX, United States

**Keywords:** cardiovascular diseases, arrhythmia detection, deep learning, electrocardiography, cardiology, anomaly

## Abstract

Cardiovascular diseases are a leading cause of mortality globally. Electrocardiography (ECG) still represents the benchmark approach for identifying cardiac irregularities. Automatic detection of abnormalities from the ECG can aid in the early detection, diagnosis, and prevention of cardiovascular diseases. Deep Learning (DL) architectures have been successfully employed for arrhythmia detection and classification and offered superior performance to traditional shallow Machine Learning (ML) approaches. This survey categorizes and compares the DL architectures used in ECG arrhythmia detection from 2017–2023 that have exhibited superior performance. Different DL models such as Convolutional Neural Networks (CNNs), Multilayer Perceptrons (MLPs), Transformers, and Recurrent Neural Networks (RNNs) are reviewed, and a summary of their effectiveness is provided. This survey provides a comprehensive roadmap to expedite the acclimation process for emerging researchers willing to develop efficient algorithms for detecting ECG anomalies using DL models. Our tailored guidelines bridge the knowledge gap allowing newcomers to align smoothly with the prevailing research trends in ECG arrhythmia detection. We shed light on potential areas for future research and refinement in model development and optimization, intending to stimulate advancement in ECG arrhythmia detection and classification.

## 1 Introduction

Electrocardiography (ECG) represents a benchmark tool for detecting and classifying cardiovascular diseases. ECG captures the heart’s electrical activity, making it ideal for detecting cardiovascular diseases (Koppad, 2021). While ECG is imperfect and may not be able to detect every pathology, it can often provide critical information about the heart’s condition. It is mainly used for the diagnosis of ischemic heart disease/coronary artery disease, myocardial infarction (heart attack), arrhythmias (abnormal heart rhythms), and cardiomyopathy (heart muscle disease). Given that arrhythmia is precipitated by malfunctions in the heart’s electrical system, the ECG provides a directand non-invasive mechanism to examine these conditions (Sun et al., 2021). It is non-invasive and hence presents no harm or discomfort to patients and can be repeatedly performed, facilitating continuous monitoring for extended durations. Moreover, ECG machines, like Holter monitors, are cost-effective and standard apparatuses in medical establishments, thus ensuring their wide availability. These devices offer swift, exhaustive data concerning the heart, encompassing aspects such as heart rate, rhythm, and evidence of possible pathologies. Established interpretative guidelines for ECG traces contribute to the identification of specific markers for diverse arrhythmia types (Aljohani, 2022). Furthermore, the characteristics of ECG signals, coupled with the substantial volume of ECG data obtainable, make them well suited for application and analysis *via* machine learning (ML) methodologies.

Despite the valuable role that ECG plays in arrhythmia detection and classification, the analysis of ECG faces several challenges. A traditional electrocardiographic analysis is often labor-intensive and dependent on the expertise of skilled clinicians, which may lead to interpretative discrepancies (Halford, 2009). Moreover, standard ECG machines may not capture sporadic or transient arrhythmias if they do not occur during recording, presenting a significant drawback in their sensitivity. Furthermore, the requirement for physical contact points (electrodes) and their placement could also affect the accuracy of the recordings while potentially causing discomfort to patients over extended periods. Accessibility in remote areas or resource-limited settings may also pose challenges due to the cost of equipment and the need for trained healthcare providers. Additionally, ECG signals are susceptible to noise and artifacts, such as powerline interference, muscle activity, electrode contact issues, and motion artifacts, distorting the waveform and affecting analysis accuracy. The noise contamination necessitates advanced signal processing techniques for reliable interpretation. Consequently, despite a negative ECG result, additional investigations may be warranted for a thorough medical evaluation of the patient’s possible conditions.

This sets the stage for developing and integrating automated, low-cost systems employing deep learning (DL) and machine learning models (Akkus et al., 2019). Such systems offer the potential for continuous, real-time monitoring and more accurate interpretation of ECG signals, thereby increasing the likelihood of capturing intermittent arrhythmias. The use of ML and DL models in ECG interpretation could also standardize analyses, reducing the variability inherent in human interpretation and potentially leading to improved patient outcomes (Ansari et al., 2023b). Thus, it is essential to foster research and development in the promising field of ML to realize its potential benefits fully.

DL methods have delivered promising results for a variety of applications, including computer vision (Wu et al., 2017; Ansari et al., 2023a), speech recognition (Deng and Platt, 2014), signal analysis (Gao et al., 2021), classification, image, and pixel analysis (Hausen and Robertson, 2020; Ansari et al., 2022c; Ansari and Qaraqe, 2023), risk analysis (Akhtar et al., 2021; Ansari et al., 2022b) and natural language processing (Bengio and LeCun, 2007). Most ECG interpretation algorithms employ DL methodologies, leveraging their inherent abilities to extract and process the information in ECG time series for improved detection and accurate classification. Some DL methodologies remove the need for manual feature selection and extraction, offering automatic feature selection and superior performance (Chandrasekar et al., 2023). Advancements in DL techniques and ease of availability of systems with higher computational capacity have catalyzed significant progress in arrhythmia detection and classification. This progress is fueled by DL’s inherent abilities to capture and interpret temporal variations in ECG signals. This property allows DL models to understand the different types of arrhythmias (Chu et al., 2023). Prominent DL algorithms like Recurrent Neural Networks (RNNs), Convolutional Neural Networks (CNNs), and Transformers possess the ability to understand both short-term patterns within individual heartbeats and long-term irregularities spanning multiple heartbeats (Attia et al., 2019). This property allows the detection of conditions like Premature Ventricular Contraction (PVC) and Atrial Fibrillation (AF), which depend on single heartbeats and may require pattern identification across multiple heartbeats (Khan and Kim, 2021). In cases wherein variations are required to be observed in a specific beat, like in the conditions of Premature Atrial or Ventricular Contractions, DL offers dynamic classifiers that can preserve long-term memory, which is key in solving such classification problems. Conversely, dynamic classifiers can also preserve short-term memory, which allows them to address conditions of Ectopic Beats characterized by deflection of the P-wave from its usual sinusoidal form (Mathur et al., 2020). Overall, DL exhibits inherent properties that make it ideal for rapid learning and subsequent classification of cardiac arrhythmia and its types.

Previous literature such as Bizopoulos and Koutsouris (2018), Dewangan and Shukla (2015), Dinakarrao et al. (2019), and Luz et al. (2016) have offered an overview of detection and classification methods for arrhythmia up to the year 2019. However, we identified a conspicuous void of comprehensive surveys enveloping the recent years, with only a few works like (Parvaneh et al., 2019; Teplitzky et al., 2020; Xiao et al., 2023a) not considering studies past the year 2022. These investigations have surveyed existing literature but need to improve their provision of in-depth comparative chronological analyses. To the best of our knowledge, no tutorial is tailored explicitly for novice researchers, enabling them to assimilate the current research trends in this domain quickly. This shortage has highlighted the need to focus on the highest-performing studies rather than indiscriminately collecting all existing works.

For instance (Koppad, 2021), analyzes 25 papers published from 2016 to 2020, primarily leveraging Convolutional Neural Networks in their summary and mainly using the MIT-BIH database. However, this work is not sufficiently comprehensive. Similarly (Hong et al., 2020), reviews 191 papers, predominantly published before 2019, exploring a variety of DL architectures for ECG analytics tasks, yet it needs in-depth comparative analysis. These papers need more intertextual analysis to guide novice readers.

Reviews by (Ebrahimi et al., 2020; Hammad et al., 2021) present detailed analyses of papers published within specific short periods (2017–2018). In addition (Ebrahimi et al., 2020), does not focus solely on DL, and (Hammad et al., 2021) reviews work on shockable arrhythmia detection based on shallow ML and DL methods.

Recent years have seen reviews like (Xiao et al., 2023a), which map recent DL works (up to the year 2022) quite comprehensively. However, this review is not tailored for novice researchers and is an indiscriminate compilation of DL works with little intertextual summarization.

Diverging from other studies, our work exclusively provides an introductory tutorial to enable new researchers to assimilate the necessary technical knowledge on arrhythmia and its classification. We provide insights into the performance and characteristics of DL methods and their variants for ECG arrhythmia detection from 2017 to the present. This overview highlights only the DL models with superior performance above 96% in terms of specificity, sensitivity, accuracy, and F1-score. This paper also provides an exhaustive compilation of traditionally utilized datasets to train the DL models for arrhythmia detection. Lastly, this survey establishes guidelines and pipelines tailored for novice researchers.

The main contributions of this paper are summarized next. This paper offers the following.

• A comprehensive tutorial designed to enable starting researchers to easily access all the information pertinent to ECG anomalies detection and classification.
• A thorough description of the arrhythmia disease, its types and classes used in detection and classification applications.
• An exhaustive compilation of standard datasets that are traditionally utilized to train and validate DL classifiers.
• A comparative analysis of the state-of-the-art DL models, while providing intertextual comparisons to serve as guideline for future work.
• A methodology pipeline to follow for addressing the DL classification task of ECG signals, with the intention of fostering the development of new contributions.

The rest of this review is organized as follows. In the Methods section, we describe the methodology used to conduct a thorough and unbiased assessment of the advancements achieved in deep learning for detecting and classifying arrhythmias in electrocardiograms. In the Deep Learning Techniques section, we describe the various deep learning models used in ECG signal processing. The Medical Background section provides the required medical background and knowledge of arrhythmias and their occurrences in ECG signals. The Datasets section discusses datasets traditionally used for model training and validation, emphasizing the importance of standardized and openly accessible datasets. In the Results and Discussion section, we thoroughly analyze and compare the performance of various DL algorithms from the present literature, summarizing significant findings and investigating prospective future research directions. The section Guideline presents the workflow pipeline for developing ECG arrhythmia detectors. Finally, the Conclusion section reinforces our main findings and summarizes our work.

## 2 Methods

Our systematic review was designed to critically evaluate the recent advancements in deep learning for identifying and classifying ECG arrhythmias, thus serving as a valuable resource for researchers in the field. We focused our review on studies published from January 2017 to January 2023, marked by significant advancements in DL, including introducing new models, such as transformers, that have substantially contributed to ECG arrhythmia detection and classification.

We devised a comprehensive and replicable search strategy to ensure an exhaustive and unbiased review. We identified key terms commonly found in current research studies on detecting and categorizing various arrhythmia types using DL. These included, but were not limited to, “Arrhythmia detection,” “ECG arrhythmia,” “Ventricular arrhythmias,” “Supraventricular arrhythmias,” “Premature beats,” “Heart block,” “Bradycardia,” “Tachycardia,” “12-Lead ECG”, “Cardiac signal processing,” “Deep learning in ECG,” and specific DL models such as “CNN,” “DNN,” “LSTM,” “Transformers,” and “Hybrid models.”

Our search was conducted across four significant databases: Google Scholar, PubMed, Scopus, and the Digital Bibliography and Library Project. We combined our search terms with Boolean operators to generate relevant search queries such as “Arrhythmia detection AND deep learning,” “Arrhythmia classification AND deep neural networks,” and “Ventricular arrhythmias AND convolutional neural networks OR CNN.” Our search was focused on studies published up until January 2023.

A total of 4,215 studies were initially identified, and after removing duplicates and filtering out the papers based on their titles, 2,492 unique studies remained. Out of the 2,492 unique studies, 207 were excluded due to language barriers as they were unavailable in English. A further 153 studies were excluded due to the unavailability of full text, leaving us with 2,132 studies for further screening. We screened these studies independently by abstract and conclusion sections, excluding 2013 papers that did not meet our predefined criteria. We then conducted a full-text assessment of the remaining studies, excluding 41 that did not meet our inclusion criteria; we had 78 studies. Studies were included if they were published in English, used DL for arrhythmia classification with ECG signals, and showed model performances with an accuracy rate of 96% or higher. Studies focusing on tasks other than arrhythmia detection, such as emotion detection or drug and alcohol assessment, were excluded, as were studies without available full text. This process was undertaken by two independent reviewers (YA and OM) and validated by a third (ES) to ensure unbiased results.

**FIGURE 1:**
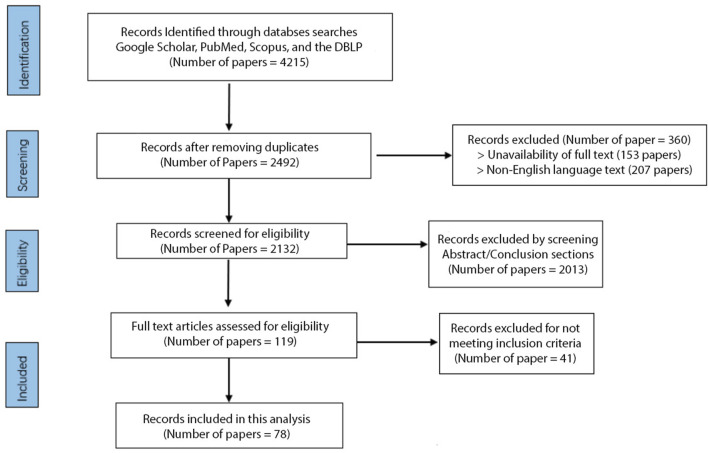
Paper search and refinement process.

At the end of this process, 78 papers representing state-of-the-art literature in the field were included in the review (Figure 1). While our systematic review protocol was not registered, our methodology is outlined clearly in this section for transparency. We conducted an extensive intertextual analysis of the selected publications to identify prevalent trends, common themes, and significant differences. This review focuses on high-performing, cutting-edge methodologies to capture an accurate snapshot of this field’s current state of research. This approach allowed us to highlight the most promising methods and recurring limitations, thereby identifying avenues for future advancement in ECG arrhythmia detection and classification using deep learning. While our review is comprehensive, a formal risk of bias assessment was not conducted for the included studies.

## 3 Deep Learning Techniques

This section presents an overview of DL methods commonly employed in ECG data analysis for arrhythmia diagnosis. Deep learning methods use artificial neural networks with multiple layers to learn hierarchical representations of data. They are instrumental in ECG analysis because they excel at extracting complicated features from raw input data. Researchers have made considerable advances in the accuracy of arrhythmia detection and classification tasks by applying DL (Parvaneh et al., 2019). In this section, we discuss fundamental DL techniques like the feedforward Multilayer Perceptron (MLP), the locally receptive Convolutional Neural Network (CNN), the sequence-aware Recurrent Neural Network (RNN), the memory-adept Long Short-Term Memory (LSTM), the simplified and efficient Gated Recurrent Unit (GRU), the generative Deep Belief Network (DBN), and the attention-based Transformer that have proven to produce excellent results to analyze ECG data. This choice of these various strategies can be explained by the distinct advantages that each one of them has in terms of recording specific ECG signal pattern characteristics. We have selected these methods because of their diverse strengths in handling various types of data and learning challenges, which provide a complete perspective of the potential of deep learning approaches in ECG interpretation. By presenting these techniques, we aim to understand better their strengths, limitations, and specialized applications in improving arrhythmia detection. Throughout this section, we discuss the principles underlying DL and emphasize how these methods might improve the accuracy and efficiency of arrhythmia analysis in clinical situations.

### 3.1 Multilayer Perceptron (MLP)

The Multilayer Perceptron (MLP) represents a variation of the artificial neural network (ANN) comprising numerous layers of interconnected nodes, commonly referred to as artificial neurons. In order to produce an output, each neuron in an MLP takes inputs, applies a weighted sum, and then passes the outcome via an activation function. This multilayer structure is particularly well suited for ECG data because it allows the model to accurately represent higher-level, nonlinear relationships within the data, which simpler models frequently struggle to do. Since MLPs can accurately simulate complicated nonlinear interactions, they are frequently used in DL approaches (Montesinos-López et al., 2021). An MLP may be used as a potent tool for arrhythmia identification and classification utilizing ECG data. The ECG data represents the heart’s electrical activity, which may be preprocessed and separated into fixed-length segments. The MLP then receives these segments as input. The MLP can handle ECG segments of various lengths and complexity levels attributable to its adaptability in input format, which enables it to manage a variety of ECG signal properties. Every input corresponds to a particular time interval or a feature taken from the ECG signal. The model can capture complex patterns related to various arrhythmias because the MLP’s hidden layers learn to extract higher-level representations and patterns from the input data. The input segment of the MLP is intended to be categorized into one of the specified arrhythmia classes by the output layer of the MLP (Özbay et al., 2006). MLP models learn to generalize and effectively categorize unknown ECG segments by being trained on a labeled dataset of ECG segments with their corresponding arrhythmia categories, making them a valuable tool for arrhythmia detection and classification in clinical situations. MLPs’ drawback is that they do not take into account the temporal connections between input data, which can be significant in the setting of ECG signals, where repeating patterns can signify certain arrhythmias.

### 3.2 Convolutional neural network (CNN)

A Convolutional Neural Network (CNN) is a class of artificial neural networks primarily used for signal analysis, image recognition, pixel data, and natural language processing. They are fit at identifying spatial hierarchies or patterns using stacked trainable small filters called kernels (Hong et al., 2020). These kernels may effectively extract local information from the context of ECG data, such as the shape and duration of heartbeats, which are essential for diagnosing arrhythmias. When working with raw ECG data, one-dimensional convolutional neural networks (1D CNNs) apply kernels along the temporal dimension (Nurmaini et al., 2020b), whereas two-dimensional convolutional neural networks (2D CNNs) deal with ECG data transformed into images and other two-dimensional formats (Ansari et al., 2021). Examples of such transformations include distance distribution matrices that are derived from entropy computations (Gabrié et al., 2018) as well as gray-level co-occurrence matrices (De Siqueira et al., 2013) and beat-to-beat correlations (Wen et al., 2019). When applied to ECG signal analysis, CNNs automatically learn and extract relevant features from raw ECG signals, improving the accuracy of arrhythmia detection. By recognizing characteristic wave patterns, CNNs can differentiate between normal and abnormal heart rhythms. However, because CNNs have a fixed receptive field size, they might have difficulties processing lengthy sequences. As a result, they might overlook long-term dependencies in the information, which are crucial for understanding ECG signals.

### 3.3 Recurrent neural network (RNN)

Recurrent Neural Networks (RNNs) stand for another class of artificial neural networks equipped with feedback mechanisms that are fit to capture temporal correlations from time series data. RNNs are equipped with cyclic connections, which sets them apart from conventional feedforward neural networks. Because of their capacity to store temporal information, RNNs are exceptionally well suited for ECG analysis, where the sequential nature of cardiac rhythms is essential for spotting anomalies. This structure allows them to retain information across time, making them appropriate for ECG data processing (Khan and Kim, 2021). The raw ECG signal must first undergo preprocessing to remove noise and undesirable artifacts before identifying and categorizing arrhythmias. The cleaned ECG data is then divided into equal-sized segments, each corresponding to a heartbeat or a specific time length. These segments are then supplied into the RNN as sequential input vectors, allowing the network to understand the ECG data’s underlying temporal dynamics. The RNN recognizes and extracts pertinent characteristics from the ECG data as it navigates through the sequence. It uses the learned features to identify and classify arrhythmias (Zhou et al., 2018). However, a typical problem with RNNs is the vanishing gradient problem, which makes it challenging to train them to understand long-term dependencies. This problem could hinder their ability to deal with lengthy ECG segments or intricate sequential patterns. As a result, the intrinsic capacity of RNNs to analyze sequential data paves the way for more accurate and robust systems for arrhythmia detection and classification, thereby playing a pivotal role in the detection and treatment of cardiovascular diseases.

### 3.4 Long Short-Term Memory (LSTM)

Long Short-Term Memory Networks (LSTMs) are a form of RNNs designed to recall data over extended periods, making them ideal for sequential data (Zhou et al., 2018). LSTMs present unique characteristics, such as memory cells that retain information and gates that govern the flow of information into and out of these cells. These gates allow LSTMs to learn and retain longer sequences, which is crucial when working with ECG data that exhibit significant properties over long time scales. These features help overcome the vanishing gradient problem common in standard RNNs. This feature of LSTMs makes them particularly useful for problems involving learning from temporal data sequences, such as ECG signals (Khan and Kim, 2021). By converting the ECG signal into sequential data segments, ECG data may be fed into an LSTM for arrhythmia detection and classification. Each segment depicts a sequence of electrical cardiac activities collected over a specific period. The time-series data, which consists of sequential cardiac cycles, is fed into the LSTM so that the interdependencies between these heartbeats may be learned and modeled. Based on this learned knowledge, the LSTM can identify and categorize arrhythmia patterns, making it a valuable tool for the automated detection of ECG cardiac arrhythmias. However, LSTMs are computationally more expensive than more straightforward models like RNNs or MLPs despite having more sophisticated features. It can be challenging to meet this increased processing requirement, mainly when working with big ECG datasets. Compared to conventional approaches, LSTMs’ unique capacity to analyze and learn from sequential data enables more precise and efficient identification and categorization of arrhythmia.

### 3.5 Gated Recurrent Unit (GRU)

Gated Recurrent Units (GRUs) are a type of RNN and were created to mitigate the vanishing gradient of standard RNNs. This feature helped to improve their capacity to capture long-term relationships in data. GRUs, which distinguish themselves by their update and reset gates, manage the flow of information by selectively remembering pertinent information and discarding irrelevant data (Esteban et al., 2016). This makes GRUs ideal for tasks that require sequential data processing and long-term dependency modeling, such as ECG signal analysis. ECG data may be divided into several time-sequenced data blocks for detecting and categorizing arrhythmias, each representing a sequence of electrical cardiac activity during a specific period. The GRU receives this time-series data, which is made up of sequential heartbeats. The GRU learns and models the temporal connections between these sequential heartbeats, permitting the detection and categorization of arrhythmic patterns based on the learned temporal features (Murat et al., 2020). GRUs, like LSTMs, are computationally intensive, which can make them difficult to use with massive ECG datasets. By utilizing GRUs’ unique capacity to analyze and interpret sequential data, it is feasible to obtain more accurate and efficient detection and categorization of cardiac arrhythmias, improving prediction performance compared to previous approaches.

### 3.6 Deep Belief Network (DBN)

Deep Belief Networks (DBNs) are a class of deep neural networks comprising several layers of latent variables or ‘hidden units’ with connections only permissible between layers, not within levels (Sarikaya et al., 2014). DBNs are typically made up of stacks of Restricted Boltzmann Machines (RBMs) or autoencoders, in which the hidden variables of each layer serve as the visible variables for the following layer. DBNs may help develop robust, discriminative models by discovering complex patterns inside datasets using the probabilistic model, which enables them to generate top-down models. They are, therefore, appropriate for applications requiring high-level data abstraction, such as identifying arrhythmia-indicating hidden patterns in ECG signals. DBNs may learn to represent ECG data in a way that captures the significant patterns or characteristics in the data, which assists in identifying irregular heartbeats and arrhythmias (Taji et al., 2017). The drawback of DBNs is that they, like other deep learning models, need a lot of labeled data for training, which can be difficult given the lack of labeled ECG datasets.

### 3.7 Transformers

Transformers stand for an effective deep learning model architecture that was first presented for natural language processing applications and has since shown promise in several other fields (Hu et al., 2022). They employ self-attention mechanisms to capture long-term dependencies and background information more successfully. Transformers equipped with attention mechanisms may be used to learn complicated patterns and connections within time-series data to identify and categorize arrhythmias in ECG data (Yan et al., 2019). By modeling each ECG signal as a sequence of data points, the transformer model can interpret the input signal by responding to critical aspects and capturing temporal relationships over the whole sequence. This property allows the model to assess both local and global patterns at the same time. Transformers are less simple to use because they require much computation and may need different hyperparameters, like attention heads and model sizes, to adjust. The transformer model uses its ability to evaluate and understand sequential data for more precise and efficient identification and categorization of cardiac arrhythmias.

## 4 Medical background

### 4.1 Arrhythmia

Arrhythmia refers to the condition of having an irregularity or anomaly in the rhythm of the heartbeat. The normal heartbeat follows a pattern known as sinus rhythm, in which electrical signals are generated by the sinoatrial (SA) node in the heart’s right atrium. These signals go through specialized routes known as the conduction system and coordinate the contraction of the heart’s chambers, resulting in a regular and synchronized beating (Antzelevitch and Burashnikov, 2011). However, various factors can disturb the regular heart-beating rhythms and lead to arrhythmias. Arrhythmias are categorized into numerous kinds according to their origin, mechanism, and characteristics. The two broad categories are tachyarrhythmias and bradyarrhythmias. Tachyarrhythmias are aberrant cardiac rhythms characterized by a rapid heartbeat, whereas bradyarrhythmias manifest through a slow heartbeat.

• **Tachyarrhythmias** is divided into supraventricular and ventricular tachyarrhythmias.
  • **Supraventricular Tachyarrhythmias** include Atrial Fibrillation (AF), Atrial Flutter, and Paroxysmal Supraventricular Tachycardia (PSVT). The most common persistent tachyarrhythmia, atrial fibrillation, is caused by disordered electrical impulses in the atria, resulting in irregular and typically fast heartbeats. Atrial flutter is identified by frequent, fast atrial contractions, as shown by a sawtooth-shaped waveform on the ECG. PSVT manifests as intermittent episodes of rapid heart rate stemming from abnormal electrical pathways or re-entry circuits in the atria or atrioventricular (AV) node.
  • **Ventricular Tachyarrhythmias** include ventricular tachycardia (VT) and ventricular fibrillation (VF). VT is characterized by a fast cardiac rhythm that originates in the ventricles and often exceeds 100 beats per minute. It happens when aberrant electrical impulses cause the ventricles to contract quicker. VF is a potentially fatal arrhythmia that causes disorganized and chaotic electrical activity inside the ventricles. If immediate care is not initiated, VF limits efficient blood pumping and can result in cardiac arrest.
• **Bradyarrhythmias** are typically caused by the conditions of Sinus Node Dysfunction or Atrioventricular (AV) conduction.
  • **Sinus Node Dysfunction** refers to a slow heart rhythm at a rate lesser than average (generally less than 60 beats per minute). It occurs due to abnormal electrical activity in the Sinoatrial (SA) Node, which is responsible for starting electrical signals in the heart.
  • **Atrioventricular (AV) Conduction Disorders** are due to anomalies in the passage of electrical impulses between the atria and ventricles. These disorders are of three types: first-degree AV block, second-degree AV block, and third-degree AV block. The second-degree AV block presents Type I and Type II subclasses, and the third-degree AV usually indicates the total heart block. First-degree AV block is characterized by a delay in electrical conduction, resulting in a longer ECG PR interval. Second-degree AV block is defined as intermittent or partial electrical conduction failure, with Type I characterized by a gradual lengthening of PR intervals until a dropped beat occurs. Type II second-degree AV block is characterized by intermittent non-conducted atrial beats without gradual PR prolongation. Third-degree AV block is characterized by a total electrical signal blockage between the atria and ventricles, frequently necessitating pacemaker installation for optimal heart rate control.

Electrocardiogram (ECG or EKG) is one of the benchmark approaches for detecting arrhythmia. An ECG analyzes the heart’s electrical activity and converts it into line tracings on paper called waves (Teich et al., 2000). An ECG scan depends on the placement of electrodes, which are small plastic patches that stick to the skin on certain spots on the patient’s chest, arms, and legs. These electrodes record the electrical signals of the patient’s heart and send them to a machine that maps the signals as waves for medical diagnosis.

### 4.2 Readings of ECG

**FIGURE 2:**
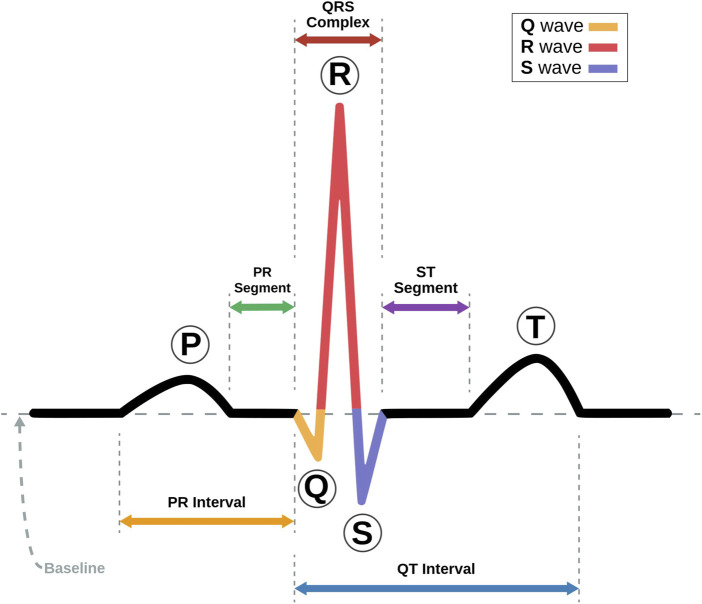
Schematic representation of an ECG Signal with its various intervals marked (Adapted with permission from Nayan and Ab Hamid, 2019).

The ECG trace is made up of five major components, each of which provides crucial information for the diagnosis of heart disorders (Teich et al., 2000). Figure 2 shows a labeled visual schematic of a standard ECG signal. The following are the five components of an ECG trace.

• **P wave** represents the atria’s depolarization and subsequent contraction. It depicts the propagation of electrical impulses across the atrial myocardium, which results in atrial contraction and the initiation of ventricular filling. Conditions including atrial enlargement, conduction problems, or atrial arrhythmias may be indicated by P waveform, duration, or amplitude anomalies.
• **QRS complex** comprises three different graphical deflections: Q, R, and S. It reflects the depolarization and subsequent contraction of the ventricles. The QRS complex reflects electrical signal transmission through the ventricular myocardium, resulting in the violent ejection of blood from the ventricles. Deviations in the duration, amplitude, or shape of the QRS complex may indicate ventricular hypertrophy, bundle branch blockages, or ventricular arrhythmias.
• **T wave** represents the ventricle’s repolarization or recovery phase. It symbolizes the ventricular myocardium being returned to its resting condition. T wave shape, amplitude, or duration changes may suggest various cardiac problems, such as myocardial ischemia, electrolyte imbalances, or drug effects.
• **U wave** is an extra wave that is seen following the T wave in some instances. It is thought to signify additional ventricular recovery. Its clear physiological relevance and clinical ramifications are currently being researched.
• **PR interval** denotes the time an electrical wave takes to travel from the atria to the AV node and then to the ventricles. It represents the time necessary to initiate atrial depolarization, atrioventricular conduction, and ventricular depolarization. Prolonged PR intervals might indicate AV conduction delays or AV blocks.
• **QT interval** represents the entire duration required for ventricular depolarization and repolarization. It denotes the duration of ventricular systole. QT interval abnormalities, particularly QT prolongation, may predispose people to potentially fatal ventricular arrhythmias.
• **ST segment** stands for the time elapsed between depolarization and repolarization of ventricles. It connects the T wave and the QRS Complex. Changes in the ST segment, such as elevation or depression, might provide important information about myocardial ischemia or damage.

A thorough comprehension of these components, intervals, and segments is required to interpret ECG results accurately. Analyzing their properties, variations, and correlations helps to diagnose various heart diseases and guides proper management and treatment techniques. Arrhythmias can be asymptomatic or severe, causing palpitations, dizziness, chest pain, shortness of breath, and possibly loss of consciousness (Abbott, 2005). Arrhythmias are frequently diagnosed and classified by examining ECG records, which give vital information on the heart’s electrical activity. Depending on the nature and degree of the arrhythmia, treatment options may include medication, electrical cardioversion, catheter ablation, or implanted devices such as pacemakers or implantable cardioverter-defibrillators (ICDs). A thorough examination and precise categorization of arrhythmias are required to develop suitable treatment strategies and provide optimal patient care.

## 5 Datasets

This section covers the ECG datasets commonly used in deep-learning models to detect cardiovascular diseases. These datasets consist of ECG signals collected from patients and are annotated with the corresponding cardiac events and conditions. These datasets have played a crucial role in developing DL algorithms for ECG signal processing, leading to notable advancements in cardiovascular disease detection.

### 5.1 Creighton university ventricular tachyarrhythmia database (CUDB)

The CUDB dataset includes 35 short-term ECG recordings of patients with sustained ventricular tachycardia, ventricular flutter, and ventricular fibrillation. Each record has 127,232 samples and was recorded for 8 minutes. Each signal employed a 12-bit resolution, encompassing a range of 10V for digitization. The signals were passed through an active second-order Bessel low-pass filter. This dataset was recorded and compiled at the Creighton University Cardiac Center and is especially pertinent for studies that try to detect and predict Ventricular Tachycardia (VT) and Ventricular Fibrillation (VF) (Li et al., 2013).

### 5.2 MIT-BIH noise stress test database (NSTDB)

The NSTDB was compiled by the Massachusetts Institute of Technology (MIT) and Boston’s Beth Israel Deaconess Medical Center (Moody et al., 1984). It records twelve 30 min long ECG recordings. It also contains three half-hour noise recordings. The ECG gathered had no element of noise, making it less practical for applications. Two noise-free recordings (numbered 118 and 119) from the MIT-BIH Arrhythmia Database were added to add noise to the data. The noise was introduced after the first 5 minutes of each record during 2-min parts alternating with 2-min clear segments. This dataset may be used to assess the resilience and reliability of arrhythmia detectors in noisy environments.

### 5.3 St petersburg INCART 12-lead arrhythmia database (INCARTDB)

The INCARTDB records 75 long-term 12-lead ECG recordings, with each recording having a duration of 30 min. The signals were sampled at 257 Hz. The data was collected from 17 male and 15 female patients, all between the ages of 18 and 80 years. This dataset records data of patients undergoing tests for coronary conduit illness. While no patients had an implanted pacemaker, the majority had ventricular ectopic beats. St. Petersburg Institute of Cardiological Technics (Incart), St. Petersburg, Russia, compiled this dataset, and it serves as a standard dataset for multiclass arrhythmia characterization (Tihonenko et al., 2008).

### 5.4 Long-term AF database (LTAFDB)

The LTAFDB dataset records 84 long-term two-lead ECG recordings of subjects with paroxysmal or supported Atrial Fibrillation (AF) conditions. Each recording had a duration between 24 and 25 h. Each signal was sampled at 128 Hz, employing a 12-bit resolution, encompassed a range of 20 mV for digitization. Two prominent annotations are available for the ECG; a computerized QRS identifier created the QRS annotations, and the ATR annotations were manually edited through a mechanized ECG examination framework. This dataset was compiled by Boston’s Beth Israel Deaconess Medical Center.

### 5.5 MIT-BIH arrhythmia database

This dataset is the most utilized dataset for detecting and classifying arrhythmia (Moody and Mark, 2001). It records 48 two-lead ECG recordings collected from 47 subjects by the BIH Arrhythmia Research facility. Each ECG recording is 30 min long, and was sampled at 360 samples/second, employing an 11-bit resolution, encompassing a range of 10 mV for digitization. This dataset was compiled by the Massachusetts Institute of Technology (MIT) and Boston’s Beth Israel Deaconess Medical Center.

### 5.6 MIT-BIH atrial fibrillation database

This dataset records data from 25 patients with Atrial Fibrillation conditions. Each ECG record is 10 h in duration and has two signals. Each signal is sampled at 250 samples/second, employing a 12-bit resolution, encompassing a range of 10 mV. Ambulatory ECG recorders with a standard recording bandwidth of 0.1 Hz–40 Hz were used to produce the recordings. This database serves as a standard database for Atrial Fibrillation detection and classification (Moody and Mark, 2001).

### 5.7 MIT BIH Normal Sinus Rhythm database

This dataset records eighteen 2-lead ECG recordings. The patients for this database had no significant arrhythmias and were aged between 20 and 50 years, with 5 being men and 13 being women. It serves as an authoritative database for detecting and classifying Normal Sinus heartbeats (Moody and Mark, 2001).

### 5.8 MIT-BIH malignant Ventricular Ectopy database

This database includes twenty-two 12-lead ECG readings. Each reading has a duration of 30 min. It records data of patients who experienced sustained ventricular tachycardia, ventricular flutter, and ventricular fibrillation episodes. These recordings are only rhythm annotated. They serve as the standard database for training DL models to detect and classify the Ventricular Ectopy class of heartbeats (Moody and Mark, 1990).

### 5.9 MIT-BIH supraventricular arrhythmia database (SVDB)

The SVDB dataset enriches the MIT-BIH Arrhythmia Database better to handle the Supraventricular (SV) arrhythmias class. It holds 78 ECG recordings, each 30 min in duration. Each recording contains two signals sampled at 250 samples/second, employing an 11-bit resolution for digitization. For annotation, the database includes symbols marking the points where heartbeats begin (the R wave of the QRS complex) and symbols indicating the beat type. In addition, the records include rhythm and signal quality annotations. This dataset is well suited for training DL models to detect Supraventricular (SV) arrhythmias (Moody and Mark, 1990).

### 5.10 Sudden Cardiac Death holter database (SCDDB)

The SCDDB holds 23 recordings from 18 people with prior conditions of prolonged Ventricular Tachyarrhythmia (VT), Ventricular Fibrillation (VF), or Cardiac Death. The recordings present in SCDDB are all snippets from lengthier ECG recordings. This dataset contains data from 18 individuals with underlying sinus rhythm conditions, four with subjects with intermittent pacing, one with continuous pacing, and four with Atrial Fibrillation (AF). Most patients whose data is recorded had experienced a confirmed cardiac arrest, and all patients had a persistent ventricular tachyarrhythmia complication. This dataset was recorded from several Boston area hospitals around the 1980s.

### 5.11 Normal Sinus Rhythm RR interval database (NSRDB)

The NSRDB dataset was compiled by the Washington University School of Medicine, St. Louis, and Rochelle Goldsmith of Columbia-Presbyterian Medical Center, New York. This dataset serves as a standard dataset for a ‘control’ group testing of arrhythmia detectors. It complies with data from eleven male participants aged between 26 and 45 years and seven female participants aged between 20 and 50 years. The dataset presents eighteen long-term ECG records of patients with no major arrhythmias complications. The ECG recordings were digitalized at 128 samples/second. This database is one of the most standard Normal Sinus Rhythm (NSR) detection and classification databases.

### 5.12 Georgia 12-lead ECG Challenge Database (GA12ECG)

The Georgia 12-Lead ECG Challenge Database was compiled at Emory University, Atlanta, Georgia, United States. This dataset contains data from 10,129 patients, producing 10,330 12-lead ECGs, classified into nine categories based on the dominant rhythm. The recordings were gathered from 5,551 male patients and 4,793 females. The recordings were sampled at 500 Hz and were recorded for 10 s each. This dataset is widely used for its large number of data points and patient pool.

### 5.13 Apnea-ECG database

Phillips University, Marburg, Germany, compiled the Apnea-ECG Database (Penzel et al., 2000). It has a total of 70 records split as follows 35 records for learning and 35 records for testing. The length of each recording ranges from 7 h to about 10 h. Each registration consists of a continuously digitalized ECG signal, a collection of apnea annotations (conducted by human specialists from respiratory and associated signals), and a group of machine-created QRS annotations containing 70 records with an average duration of 8 h from people with chances of sleep apnea conditions. An ECG signal and a breathing signal with apnea annotation are also included in every record.

### 5.14 PTB diagnostic ECG database

Physikalisch-Technische Bundesanstalt (PTB), the National Metrology Institute of Germany, compiled the PTB Diagnostic ECG Database (Flores et al., 2018). It contains 549 records from 290 subjects (209 males and 81 females). The patients selected were aged 17 to 87, with the mean age being 57.2. Each record incorporates fifteen data values that were simultaneously collected. The fifteen data values are from the regular 12 leads and the leftover 3 Frank lead ECGs. Each signal is sampled at 1,000 samples/second, employing a 16-bit resolution, encompassing a range of ±16.384 mV for digitization. On unique solicitation to the patrons of the information base, accounts might be accessible at testing rates up to 10 KHz. This dataset is suitable for a wide range of tasks, from arrhythmia detection, automated diagnosis of heart conditions, and signal quality assessment to anomaly detection.

### 5.15 European ST-T database

The CNR Institute for Clinical Physiology at Pisa and the European Society of Cardiology compiled the European ST-T dataset (Taddei et al., 1992). This dataset contains 90 ECG signal records to assess ischemic coronary illness and various arrhythmias and is used to examine ST and T-wave changes in ECG. The database holds data gathered from seventy male participants aged between 30 and 84 years and eight female participants aged between 55 and 71. The dataset holds 401 T-waves and 367 ST segment episode changes. Each episode records over 30 s, with peak displacements ranging from 100 μV to 1 mV. Each record is 120 min long, with two signals sampled at 250 samples/second, quantized with 12 bits over a nominal 20 mV input range.

### 5.16 PhysioNet computing in cardiology challenge 2017 (AFDB)

The AFDB dataset comprises 12,186 single lead ECG recordings of 30 and 60 s long, gathered from subjects undergoing long-haul mobile ECG checking. The dataset is divided into a training set of 8,528 records and 3,658 as a test set. The dataset was compiled by PhysioNet, an online database repository, using the AliveCor healthcare device. The recordings were digitized continuously at 44.1 kHz and 24-bit goal utilizing programming demodulation. At last, the recordings were put away as 300 Hz, 16-bit records with a transfer speed of 0.5–40 Hz and a ±5 mV dynamic reach. This dataset is rich for classifying different types of irregular heart rhythms (arrhythmias) from single-lead ECGs (Andreotti et al., 2017).

### 5.17 China physiological signal challenge 2018 (CPSC 2018)

The China Physiological Signal Challenge 2018 (CPSC 2018) dataset (Liu et al., 2018), compiled by 11 hospitals across China, encompasses 6,877 ECG recordings from a diverse group of subjects, including 3,699 males and 3,178 females. The recordings, which vary in length from 6 to 60 s, are stored as MAT files, with accompanying hea files providing labels and pertinent ECG recording information. Each ECG recording is sampled at a frequency of 500 Hz. The dataset is multi-labeled, with ECG recordings representing nine distinct cardiac states, including Atrial Fibrillation (AF), Intrinsic Paroxysmal Atrioventricular Block, Left Bundle Branch Block (LBBB), Normal Heartbeat, Premature Atrial Contraction (PAC), Premature Ventricular Contraction (PVC), Right Bundle Branch Block (RBBB), ST-segment Depression (STD), and ST-segment Elevation (STE). Notably, 476 of the recordings have two or three different labels. This dataset serves as a valuable resource for the development and evaluation of algorithms for rhythm and morphology abnormality detection from 12-lead ECGs. Its diverse and comprehensive nature makes it particularly suitable for research in automated diagnosis of heart conditions, signal quality assessment, and anomaly detection.

These datasets (Table 1) provide a comprehensive list of the standard databases implemented for model training for ECG arrhythmia detection and classification. The Association for the Advancement of Medical Instrumentation (AAMI) recommends training and detecting only a few types of arrhythmia. It recommends using 15 classes for arrhythmia for model training. These 15 classes are classified into five superclasses: Normal (N), SupraVentricular Ectopic Beat (SVEB), Ventricular Ectopic Beat (VEB), Fusion beat (F), and Unknown beat (Q). Table 2 presents these superclasses. Most databases presented above stand as publicly available standard datasets that have been used to train and validate high-performance DL-based classification models. These models will be discussed in the next section.

**TABLE 1:** Summary of standard databases for arrhythmia classification and detection.

Databases	Records	Leads	Time	Source	Links
The Creighton University Ventricular Tachyarrhythmia Database (CUDB)	35 records	1	8 min each	Creighton University Cardiac Center	https://archive.physionet.org/physiobank/database/cudb/
MIT-BIH Noise Stress Test Database (NSTDB)	12 records	2	30 min each	Massachusetts Institute of Technology (MIT) + Boston’s Beth Israel Deaconess Medical Center	https://physionet.org/content/nstdb/1.0.0/
St Petersburg INCART 12-lead Arrhythmia Database (INCARTDB)	32 records	12	30 min each	St. Petersburg Institute of Cardiological Technics (Incart), St. Petersburg, Russia	https://physionet.org/content/incartdb/1.0.0/
Long-Term AF Database (LTAFDB)	84 records	2	24–25 h each	Boston’s Beth Israel Deaconess Medical Center	https://physionet.org/content/ltafdb/1.0.0/
MIT-BIH Supraventricular Arrhythmia Database (SVDB)	78 records	3	30 min each	Massachusetts Institute of Technology (MIT)	https://physionet.org/content/svdb/1.0.0/
Sudden Cardiac Death Holter Database (SCDDB)	23 records	2–3	24–48 h each	Boston area hospitals	https://physionet.org/content/sddb/1.0.0/
Normal Sinus Rhythm RR Interval Database (NSRDB)	18 records	NA	24 h each	Washington University School of Medicine + Columbia-Presbyterian Medical Center	https://physionet.org/content/nsr2db/1.0.0/
Georgia 12-Lead ECG Challenge Database (GA12ECG)	20,672 records	12	5–10 s each	Emory University, Atlanta, Georgia, United States of America	https://shorturl.at/qCN79
Apnea-ECG Database	70 records	1	7–10 h each	Phillips-University, Marburg, Germany	https://physionet.org/content/apnea-ecg/1.0.0/
MIT-BIH Arrhythmia Database	48 records	3	23–48 s each	Massachusetts Institute of Technology (MIT) + Boston’s Beth Israel Deaconess Medical Center	https://www.physionet.org/content/mitdb/1.0.0/
PTB Diagnostic ECG Database	549 records	12	10 s each	Physikalisch-Technische Bundesanstalt (PTB), the National Metrology Institute of Germany	https://www.physionet.org/content/ptbdb/1.0.0/
European ST-T Database	90 records	2	2 h each	CNR Institute for Clinical Physiology + European Society of Cardiology	https://physionet.org/content/edb/1.0.0/
The PhysioNet Computing in Cardiology Challenge 2017 (AFDB)	12,186 records	1	30–60 s each	Compiled by PhysioNet aliveCor healthcare device	https://physionet.org/content/challenge-2017/1.0.0/
MITBIH Atrial Fibrillation Database	25 records	2	10 h each	Massachusetts Institute of Technology (MIT) + Boston’s Beth Israel Deaconess Medical Center	https://physionet.org/content/afdb/1.0.0/
MIT BIH Normal Sinus Rhythm Database	18 records	2	24 h each	Massachusetts Institute of Technology (MIT) + Boston’s Beth Israel Deaconess Medical Center	https://physionet.org/content/nsrdb/1.0.0/
MIT BIH Malignant Ventricular Ectopy Database	22 records	2	30 min each	Massachusetts Institute of Technology (MIT) + Boston’s Beth Israel Deaconess Medical Center	https://physionet.org/content/vfdb/1.0.0/
China Physiological Signal Challenge 2018 (CPSC 2018)	6,877 records	12	6–60 s each	11 hospitals across China	https://www.kaggle.com/datasets/bjoernjostein/china-physiological-signal-challenge-in-2018

**TABLE 2:** Classes of ECG Signals used to training.

Superclass		
N	SVEB	VEB
**(Nou.)** Normal Beat	**(A)** Atrial Premature Beat	**(V)** Premature Ventricular Contraction
**(L)** Left Bundle Branch Block Beat	**(a)** Aberrated Atrial Premature Beat	**(E)** Ventricular Escape Beat
**(R)** Right Bundle Branch Block Beat	**(J)** Nodal (Junctional) Premature Beat	
**(e)** Atrial Escape Beat	**S** Supraventricular Premature Beat	
**(j)**Nodal (Junctional) Escape Beat		

Superclass		
F		VEB
**(F)** Fusion of Ventricular and Normal Beat		**(Pou)** Paced Beat
		**(f)** Fusion of Paced and Normal Beat
		**(U)** Unclassified Beat

## 6 Results and discussion

This section provides a comprehensive intertextual summarization of some of the best-performing recent DL models for ECG arrhythmia detection and classification from 2017 to present. We discuss DL models demonstrating high overall performance, specifically those achieving over 96% in terms of accuracy, sensitivity, specificity, and F1-score. To facilitate the understanding of these models and to gauge their performances, we present two tables: Tables 3, 4.

**TABLE 3:** Summary of deep learning models for ECG arrhythmia detection and classification.

Study	Database	# Cl	Classifier	Acc (%)	Se (%)	Sp (%)
Luo et al. (2017)	MIT-BIH	4	DNN-SDA	98.80	71.40	99.80
Majumdar and Ward (2017)	MIT-BIH	4	SVM-RBF	97.00	100.0	90.12
Zhang et al. (2017)	MIT-BIH	5	RNN	99.40	97.60	99.70
Xia et al. (2017)	MIT-BIH	3	CNN	98.63	98.79	97.87
Nguyen et al. (2018)	CUDB MIT-BIH (VFDB)	2	FCN	99.26	97.07	99.44
Jun et al. (2018)	MIT-BIH	4	2D CNN	99.05	99.57	97.85
Yildirim (2018)	MIT-BIH	4	Bi-directional LSTM	99.39	95.66	98.11
Sannino and De Pietro (2018)	MIT-BIH	4	DNN	99.68	99.48	99.83
Faust et al. (2018)	MIT-BIH	5	BiLSTM	98.51	98.32	98.67
Xia and Xie (2019)	MIT-BIH	4	1D CNN + Active Learning	99.20	95.73	98.73
Lui and Chow (2018)	MIT-BIH	4	ML-CNN	96.00	95.40	97.37
Xia et al. (2018)	MIT-BIH Wearable Device	4	DNN	99.80	99.40	99.90
Wang et al. (2019)	MIT-BIH	2	GRNN	97.40	86.70	98.30
Hanbay (2019)	MIT-BIH	4	DNN	96.40	86.41	96.41
Wang and Zhou (2019)	BIDMC-CHF + MIT-BIH NSR + Fantasia	5	LSTM	99.22	99.22	99.72
Chen et al. (2020)	MIT-BIH	4	CNN-LSTM	99.32	97.50	98.70
Fu et al. (2020)	PTB	6	CNN-BiGRUt	99.11	99.02	98.23
Sharma et al. (2021)	MIT-BIH	5	SVM + FFBPNN	98.53	98.24	95.68
Ojha et al. (2022)	MIT-BIH	4	CNN-SVM	99.53	98.24	97.58
Sepahvand and Abdali-Mohammadi (2022)	Chapman ECG DB	12	Distilled Models	98.15	97.11	98.45
Midani et al. (2023)	MIT-BIH	5	CNN + BiLSTM	99.46	97.01	99.57
Kumar et al. (2023)	MIT-BIH	5	Fuzz-ClustNet	98.66	98.92	93.88

**TABLE 4:** F1-scores of deep learning models for ECG arrhythmia detection and classification.

Study	Database	# Cl	Classifier	F1-Score(%)
Luz et al. (2016)	MIT-BIH	5	GRNN	99.00
Sujadevi et al. (2017)	MIT-BIH	4	GRU	99.99
Faust et al. (2018)	MIT-BIH	5	BiLSTM	98.00
Tan et al. (2018)	Fantasia + INCARTDB	2	CNN-LSTM	99.52
Xiang et al. (2018)	MIT-BIH	8	1D CNN	99.99
Hammad et al. (2020)	MIT-BIH	5	DNN	95.30
Mahmud et al. (2020)	MIT-BIH	5	1D CNN	99.10
Ullah et al. (2020)	MIT-BIH	8	2D CNN	98.00
Peimankar and Puthusserypady (2021)	QT DB	4	CNN-LSTM	99.56
Islam et al. (2022)	MIT-BIH	5	BiGRU + BiLSTM	98.41
Hong et al. (2022)	MIT-BIH	4	ECG Delineator	96.11
Zahid et al. (2022)	MIT-BIH	5	1D Self ONN	99.51
Kim et al. (2022)	MIT-BIH	5	ResNet + BiLSTM	99.20
Sepahvand and Abdali-Mohammadi (2022)	Chapman ECG DB	12	Distilled Models	97.55
Midani et al. (2023)	MIT-BIH	5	CNN + BiLSTM	97.63
Kumar et al. (2023)	MIT-BIH	5	Fuzz-ClustNet	96.34

Table 3 comprehensively summarizes the best DL models within the selected time frame. Column 3, abbreviated as #Cl., refers to the number of arrhythmia classes considered in the multi-class detection and classification model. This table also offers a chronological summary of three performance metrics: Accuracy (Acc), Sensitivity (Se), and Specificity (Sp) for each DL classifier.

Accuracy (Acc) is an essential performance measure in classification problems. It assesses a model’s overall accuracy by computing the proportion of total predictions the model correctly predicted, including positives (arrhythmia) and negatives (no arrhythmia). While accuracy gives a rapid overview of how well a model performs, it does not provide precise details about how well it performs on particular classes, which is especially important when the dataset is imbalanced.

Sensitivity (Se), or the True Positive Rate (TPR), is the proportion of actual positives (arrhythmias) correctly recognized by the model. It is critical in medical diagnostics since high sensitivity suggests the model can reliably detect arrhythmias, lowering the possibility of false negatives. A model with inadequate sensitivity may fail to detect critical abnormalities, potentially resulting in serious health consequences.

Specificity (Sp), also known as the True Negative Rate (TNR), is the proportion of real negatives (no arrhythmias) that are accurately detected. In essence, it indicates the model’s ability to prevent incorrect diagnosis. This property is significant because a poor specificity model may result in unnecessary treatments or tests due to many false positives. This metric is important when gauging the overall reliability of a model.

Taken together, these three metrics: accuracy, sensitivity, and specificity, provide a more holistic and nuanced view of the performance of a DL model in the context of ECG arrhythmia detection and classification. They provide a balanced evaluation that accounts for overall performance and the accuracy of class identification, ensuring that the model performs well across all categories and does not overlook any one category.

The DL models proposed for ECG signal classification have adopted different pathways, each with distinctive features and limitations. Several approaches rely on the notion of patient-specific models for improved accuracy (Luo et al., 2017). employs an automatic feature abstraction and a deep neural network classifier to detect and classify arrhythmia. However, their approach requires extensive computation and other individually annotated beats as inputs (Zhang et al., 2017). shares this limitation and proposes a model based on RNNs and a density-based clustering technique limited by the unbalanced classes in the dataset. On the other hand (Majumdar and Ward, 2017), proposed a method called robust deep dictionary learning, and Xia et al. (Xia et al., 2017) adopted the stationary wavelet transform for ECG preprocessing to fit the requirements of deep convolution neural networks. These methods, however, are challenged by the long feature extraction time and dependency on the accuracy of peak detection.

Innovative implementations such as the use of ECG images for classification (Jun et al., 2018), bidirectional LSTM networks (Yildirim, 2018), and hybrid combinations of RNNs and CNNs (Lui and Chow, 2018) have also delivered promising results. However, these innovative methods need help integrating their technologies into a comprehensive system, dealing with the time cost of the training phase and possible overfitting due to small sample sizes restrained by their choice of dataset.

Meanwhile (Nguyen et al., 2018), focuses on Software Composition Analysis (SCA) detection using a CNN for feature extraction and a boosting classifier. In contrast (Sannino and De Pietro, 2018), propose a Deep Neural Network for abnormal ECG beat classification. They have delivered impressive results using minimal DL models consisting of only seven low-complexity layers. As stated by most of the works, they claim improved model performance using additional, balanced datasets.

Several works focus on detecting Atrial Fibrillation (AF), one of the significant occurring types of arrhythmias, as in the work of (Faust et al., 2018), where a hybrid DL system merged with an LSTM model was used on Heart Rate (HR) signals. This approach is promising but suffers from instrumentation limitations. Moreover (Wang et al., 2019), propose a globally applicable and updatable classification model, the Global Recurrent Neural Network (GRNN), which used active learning to learn informative beats and enlarged its training set to improve its performance.

There have also been works aimed at integrating wearable technologies for arrhythmia detection and classification, as seen in (Xia and Xie, 2019), who introduced a wireless wearable ECG device that combines an ECG acquisition device and ECG classification method in a CNN framework. However, despite the promising performance, the primary challenges in their model include computational intensity and the necessity for R-peaks detection. The development of wearable ECG technology has been significant, but bottlenecks such as the need for high precision, low power consumption, and efficient systems persist. The future prospects for wearable ECG learning involve addressing these challenges and further optimizing the technology for real-time, efficient, and precise disease warning.

An active deep learning-based classification method is proposed by (Hanbay, 2019). This work determines six statistical features for each heartbeat and combines them with the eigenvalues of ECG beats. Despite the novelty, a notable drawback is the time-consuming feature learning phase and the effect of temporal waveform patterns on feature extraction.

Similar research on ECG classification was carried out by (Chen et al., 2020), where the authors integrated CNN and LSTM models to identify six types of ECG signals. The increased diversity of subjects in the training data helped to achieve a high accuracy rate. Nevertheless, their approach requires QRS detection, adding to the computational cost, and utilizes an imbalanced dataset, which led to low classification accuracy for the atrial flutter (AFL) category.

On a different perspective (Wang and Zhou, 2019; Sharma et al., 2021), focused on healthcare applications and heartbeat abnormalities, respectively (Wang and Zhou, 2019). used short-term HRV in conjunction with mobile devices for monitoring patients’ health but acknowledged the need to address data imbalance issues in future work. Meanwhile (Sharma et al., 2021), proposed an efficient hybrid approach for classifying ECG samples into crucial arrhythmia classes, suggesting that their work could be extended to cover more arrhythmia classes in the future.

Several other works have sought to improve ECG signal classification using sophisticated models. For instance (Ojha et al., 2022), implemented a 1D-CNN model based on an Auto-encoder Convolution Network (ACN). Similarly (Sepahvand and Abdali-Mohammadi, 2022), utilized knowledge distillation for arrhythmia classification. Implementing distillation models in this field is novel and shows promise. However, in this work, the choice of the CNN model and hyperparameters may not be optimal.

A recent study by (Midani et al., 2023) suggests a novel methodological approach that combines feed-forward and recurrent deep neural networks using a sequential fusion method. This approach aims to better represent relevant features of arrhythmia in ECG signals. However, their method relied on a small database for training and testing and used R peak segmentation based on dataset annotation (Kumar et al., 2023). puts forward a technique called Fuzz-ClustNet, which combines deep learning and fuzzy clustering for detecting arrhythmia. This method relies on denoising, augmentation, and segmentation.

Despite the promising results, these studies face numerous limitations and challenges, including the need for extra individually annotated beats, class unbalances, long feature extraction time, under-representation of specific ECG beat types, limited access to rich databases, the time cost of the training phase, dependence on the accuracy of peak detection, and issues with unnecessary defibrillation. These limitations highlight the need for more robust and accurate DL models for ECG signal classification.

Table 4 extends the analysis by presenting the F1 Scores of the methods. The F1 score is a widely used measure that considers precision and recall, providing a balanced assessment of model performance. Like Table 3, the models in Table 4 also include the #Cl. Column indicating the number of arrhythmia classes handled by the model are categorized based on the 96% cutoff. Incorporating the F1 score in our analysis enhances our understanding of the model’s effectiveness in accurately identifying and classifying arrhythmias. By integrating the information from both tables, we aim to offer a holistic overview of the significant strides made by DL models in this domain.

Table 4 further explores DL methods with promising results for ECG arrhythmia detection and classification. Research has explored various methods for detecting arrhythmia, including Fuzz-ClustNet, fuzzy clustering, and a deep learning framework (Kumar et al., 2023). One approach, called ‘DeepArr,’ combines feed-forward and recurrent neural networks to improve accuracy (Midani et al., 2023). Both methods, however, are bound by certain limitations. While Fuzz-ClustNet could benefit from more sophisticated signal processing methods, DeepArr’s reliance on the small MIT-BIH arrhythmia database for training and testing reduces its generalizability.

These limitations resonate with the issues encountered in other studies, such as the works of (Kim et al., 2022; Sepahvand and Abdali-Mohammadi, 2022), which leverage advanced DL models for ECG classification but admit potential shortcomings in their design, specifically in the optimal selection of layers, filters, and hyperparameters in the former and the generalization capability of the latter.

A similar narrative is carried forward in the studies of (Hong et al., 2022; Zahid et al., 2022). Zahid et al. introduced a 1D Self-Organizing Neural Network (ONN) for ECG classification. The goal is to learn morphological representations from ECG data automatically, but the authors also recognize the possibility of enhancing the model’s complexity. Hong et al.‘s clinical ECG interpreter, in the meantime, grapples with the issue of ECG data acquisition and heterogeneous data formats, a problem akin to the lack of benchmark datasets lamented by (Islam et al., 2022) in their RNN-based arrhythmia classification study.

While (Ullah et al., 2020; Peimankar and Puthusserypady, 2021) have successfully proposed DL models that eliminate the feature engineering step and extend ECG classification to more classes, they also echo the recurring theme of ECG waveform delineation challenges and the necessity of a more extensive and more diverse dataset. This sentiment of data insufficiency extends to DeepArrNet (Mahmud et al., 2020) and multitier DNN (Hammad et al., 2020), with the latter indicating the method’s susceptibility to noisy signals and data-intensiveness.

Intertextual analysis of the gathered literature provides pivotal insights into current research trajectories and their respective constraints. Established works indicate that the limitations imposed by the lack of size and diversity in the databases surpass those dictated by the choice of learning algorithm (Torralba and Efros, 2011). The need for more extensive and more balanced datasets and efficient, less time-consuming models resonates across the research landscape. Examining the key themes emerging from the literature underscores the critical role of data quality, the utilization of public ECG databases, and the issue of data imbalance in ECG databases.

First, data quality is critical for attaining excellent classification performance, as deep learning models rely primarily on robust and diverse training data. Access to various ECG data, including all genders, ages, and health problems, is essential. The dependence on public ECG datasets, although necessary at present, has several limitations, with the need for more diverse data samples being apparent for improving the models’ clinical applicability.

Second, while the MITDB database serves as a comparison baseline for new and existing deep learning approaches, model complexity is rising, necessitating more comprehensive ECG data for practical training. The recent trend of merging data from several public ECG databases demonstrates this requirement, even though it necessitates carefully evaluating variances in patient demographics, measurement circumstances, and signal characteristics.

Finally, a significant imbalance in data categories in current arrhythmia-related ECG databases, with an overrepresentation of ‘normal’ categories, presents an additional hurdle. Although numerous approaches, including data augmentation (Ma et al., 2022) and focal loss (Li et al., 2022), have been used to solve this problem, acquiring new data in the aberrant categories is the most practical answer. However, the practical problems connected with this approach, such as the availability of particular patient groups, highlight that the imbalance in ECG datasets remains a formidable long-term challenge for researchers (Nurmaini et al., 2020a). Together, these characteristics provide insight into the complexities of the ECG arrhythmia classification process and indicate areas where further effort and innovation are needed to advance the field.

The task of arrhythmia detection and classification represents a multifaceted multiclass classification problem. Studies utilizing databases such as MIT-BIH, CUDB, and AFDB focused on the classification of the N, SVEB, VEB, F, and Q superclasses (refer to Table 2) and detection of significant types of arrhythmias like Atrial Flutter, Atrial Fibrillation (AF), and Ventricular Fibrillation. In contrast, studies utilizing PhysioNet/CinC Challenge datasets focused on the classification and detection of AF, NSR, and other rhythms by taking noise into account. Furthermore, several studies underline the possible impact of patient-specific characteristics on arrhythmia types, including age and gender, emphasizing the significance of tailored detection and classification methodologies (Haleem et al., 2021). As a result, this broader perspective on arrhythmia classification research emphasizes the multifaceted nature of the problem and the need for tailored solutions.

Traditionally, CNNs are considered the top DL models for ECG classification due to their exceptional feature extraction abilities (Ansari et al., 2022a). Meanwhile, RNNs have also shown great potential in this area by catering to the time series nature of ECG signals. The advent of transformers equipped with attention mechanisms represents a significant progress in DL models, with initial studies showing promising results.

Performance analysis of the compiled research shows that hybrid DL models, specifically transformers, perform better than traditional shallow DL models in classifying ECG arrhythmias. Most hybrid DL models for arrhythmia classification use CNNs as the first feature extractors, followed by more precise feature extraction using additional DL structures such as RNNs and transformers (Zhu et al., 2022). While these hybrid models excel in classification, they increase computational complexity (Tan et al., 2018; Oh and Lee, 2022), which is a concern. Currently, the research emphasizes combining newer DL models or structures for arrhythmia classification, such as Vision Transformers (ViT) (Han et al., 2022) and MLPMixer (Tolstikhin et al., 2021) with traditional DL models. However, this trend is in its early stages.

In addition, the focus on enhancing classification accuracy overlooks the importance of having interpretable DL models. Such models offer clarity to the ECG classification outcomes and could be highly beneficial in clinical situations. They can help better diagnose heart irregularities and uncover concealed ECG signal characteristics. Incorporating DL models into other artificial intelligence frameworks like active learning (He et al., 2022) and reinforcement learning (Xiao et al., 2023b) might considerably improve ECG diagnostic accuracy. Furthermore, systematic optimization of DL model architectures, such as convolutional kernel sizes and hyperparameters, such as minibatch size and learning rate, should improve ECG classification efficiency.

When dealing with an increased number of classification categories, the mapping relationship’s complexity and data scarcity in minority categories presents formidable challenges (Zhao et al., 2022). Innovative strategies such as the Hybrid Attention-Based Deep Learning Network (HADLN) (Jiang et al., 2021), a depthwise separable convolutional neural network with focal loss (Lu et al., 2021), and a novel approach for atrial fibrillation classification based on the 2D representation of minimal subset ECG (Zhang et al., 2023) have shown promise in addressing these challenges. Exploring such strategies and pushing the bounds of hybrid models might enhance the learning capabilities of the DL process, allowing for correct classification performance for even more categories. The path towards improving the state-of-the-art in ECG arrhythmia classification and detection seems to be through constant innovation, integrating novel methods, and optimizing existing ones.

## 7 Guideline

We propose a comprehensive, systematic, and standardized workflow pipeline that is instrumental in furthering research activities, addressing prior limitations, and standardizing the clinical evaluation process. This pipeline serves as a cardinal guide for researchers when developing and evaluating deep learning (DL) models specifically for heartbeat arrhythmia classification. It is crucial to stress that these recommendations work best in heartbeat classification settings when feature extraction and classification are carried out in discrete steps. Different issues may apply, requiring a changed strategy, for detection systems incorporating arrhythmias like AF or VF, which require segments as input and permit end-to-end learning.

1. Database selection stage:
  • Use the standard MIT-BIH database, which includes the suggested standard metrics. This choice allows for impartial comparisons with previous studies.
  • Examine the model’s generalization capabilities by including the INCART database into the assessment procedure (Llamedo and Martínez, 2010).
2. Preprocessing stage:
  • Use standard signal filtering techniques to permit direct comparisons with current literature.
  • Employ the unfiltered raw signal as the ground truth to correctly assess the model’s performance.
3. Segmentation stage:
  • Introduce jitter to the R-location annotation throughout the assessment procedure to measure the model’s robustness (Llamedo and Martínez, 2010).
4. Feature extraction stage:
  • Utilize state-of-the-art feature selectors to extract salient features (Pudil et al., 1994; Llamedo and Martínez, 2010; Mar et al., 2011; Zhang et al., 2014).
  • The use of class-oriented feature selection can provide useful insights into selecting significant features for various forms of arrhythmia (Zhang et al., 2014).
5. Classification stage:
  • Implement a k-fold cross-validation training pipeline to ensure unbiased model training.
  • Address dataset imbalances associated with certain heartbeat types by using data augmentation techniques or specialized classifiers such as LSTM networks.
6. Evaluation stage:
  • Employ standardized metrics to enable fair and unbiased comparisons between the proposed methodology and existing literature.

In summary, the extensive exploration of deep learning and machine learning techniques, combined with novel methods such as knowledge distillation and feature vector optimization, have shown encouraging results in arrhythmia detection. Despite the encountered limitations, these studies demonstrate the promise of DL methods for ECG signal classification. These innovative approaches offer novel solutions and show considerable potential for further development. Future research should address these limitations, including the development of personalized detection and classification methods, balanced and comprehensive datasets, optimal selection schemes of model parameters, and the effective incorporation of necessary components like denoising and augmentation to achieve superior performance. In addition, exploiting additional data modalities through intelligent data fusion and processing techniques capable of self-learning and adapting in real-time to a person’s specific characteristics and status remain fundamental problems. By doing so, we can more fully exploit the potential of these methods for practical application in healthcare monitoring and diagnosis.

## 8 Conclusion

Deep learning (DL) algorithms have demonstrated enormous prospects for arrhythmia detection utilizing ECG data, demonstrating the significant potential for clinical implementation. However, our review provides tailored suggestions for novice researchers to assimilate them with the necessary knowledge and trends of the field. We discuss recent research trends and address several crucial DL pipeline components that require further exploration before its clinical implementation for ECG arrhythmia categorization. We emphasize the need to focus on using various ECG databases for model training and validation and developing unique integrated DL models. These directions offer prospects for developing DL-based ECG arrhythmia classification models and encouraging their adoption in clinical practice.

## Data Availability

The original contributions presented in the study are included in the article/Supplementary material, further inquiries can be directed to the corresponding author.

## References

[B1] Abbott A. V. (2005). Diagnostic approach to palpitations. Am. Fam. physician 71, 743–750. 15742913

[B2] Akhtar Y. Dakua S. P. Abdalla A. Aboumarzouk O. M. Ansari M. Y. Abinahed J. (2021). Risk assessment of computer-aided diagnostic software for hepatic resection. IEEE Trans. Radiat. plasma Med. Sci. 6, 667–677. 10.1109/trpms.2021.3071148

[B3] Akkus Z. Cai J. Boonrod A. Zeinoddini A. Weston A. D. Philbrick K. A. (2019). A survey of deep-learning applications in ultrasound: artificial intelligence–powered ultrasound for improving clinical workflow. J. Am. Coll. Radiology 16, 1318–1328. 10.1016/j.jacr.2019.06.004 31492410

[B4] Aljohani M. S. (2022). Competency in ECG interpretation and arrhythmias management among critical care nurses in Saudi arabia: A cross sectional study. Healthcare 10, 2576. 10.3390/healthcare10122576 36554100 PMC9777912

[B5] Andreotti F. Carr O. Pimentel M. A. Mahdi A. De Vos M. (2017). “Comparing feature-based classifiers and convolutional neural networks to detect arrhythmia from short segments of ECG,” in 2017 Computing in Cardiology (CinC), France, 24-27 September 2017.

[B6] Ansari M. Y. Abdalla A. Ansari M. Y. Ansari M. I. Malluhi B. Mohanty S. (2022a). Practical utility of liver segmentation methods in clinical surgeries and interventions. BMC Med. imaging 22, 97–17. 10.1186/s12880-022-00825-2 35610600 PMC9128093

[B7] Ansari M. Y. Chandrasekar V. Singh A. V. Dakua S. P. (2022b). Re-Routing drugs to blood brain barrier: A comprehensive analysis of machine learning approaches with fingerprint amalgamation and data balancing. IEEE Access 11, 9890–9906. 10.1109/access.2022.3233110

[B8] Ansari M. Y. Mangalote I. A. C. Masri D. Dakua S. P. (2023a). “Neural network-based fast liver ultrasound image segmentation,” in 2023 International Joint Conference on Neural Networks, Australia, 18-23 June 2023.

[B9] Ansari M. Y. Qaraqe M. (2023). Mefood: A large-scale representative benchmark of quotidian foods for the middle east. IEEE Access 11, 4589–4601. 10.1109/access.2023.3234519

[B10] Ansari M. Y. Yang Y. Balakrishnan S. Abinahed J. Al-Ansari A. Warfa M. (2022c). A lightweight neural network with multiscale feature enhancement for liver ct segmentation. Sci. Rep. 12, 14153. 10.1038/s41598-022-16828-6 35986015 PMC9391485

[B11] Ansari M. Y. Yang Y. Meher P. K. Dakua S. P. (2023b). Dense-PSP-UNet: A neural network for fast inference liver ultrasound segmentation. Comput. Biol. Med. 153, 106478. 10.1016/j.compbiomed.2022.106478 36603437

[B12] Ansari Y. Tiyal N. Flushing E. F. Razak S. (2021). “Prediction of indoor wireless coverage from 3d floor plans using deep convolutional neural networks,” in LCN, Canada, 04-07 October 2021, 435–438.

[B13] Antzelevitch C. Burashnikov A. (2011). Overview of basic mechanisms of cardiac arrhythmia. Card. Electrophysiol. Clin. 3, 23–45. 10.1016/j.ccep.2010.10.012 21892379 PMC3164530

[B14] Attia Z. I. Kapa S. Lopez-Jimenez F. McKie P. M. Ladewig D. J. Satam G. (2019). Screening for cardiac contractile dysfunction using an artificial intelligence–enabled electrocardiogram. Nat. Med. 25, 70–74. 10.1038/s41591-018-0240-2 30617318

[B15] Bengio Y. LeCun Y. (2007). Scaling learning algorithms towards AI. Large-scale Kernel Mach. 34, 1–41.

[B16] Bizopoulos P. Koutsouris D. (2018). Deep learning in cardiology. IEEE Rev. Biomed. Eng. 12, 168–193. 10.1109/RBME.2018.2885714 30530339

[B17] Chandrasekar V. Ansari M. Y. Singh A. V. Uddin S. Prabhu K. S. Dash S. (2023). Investigating the use of machine learning models to understand the drugs permeability across placenta. IEEE Access 11, 52726–52739. 10.1109/access.2023.3272987

[B18] Chen C. Hua Z. Zhang R. Liu G. Wen W. (2020). Automated arrhythmia classification based on a combination network of CNN and LSTM. Biomed. Signal Process. Control 57, 101819. 10.1016/j.bspc.2019.101819

[B19] Chu M. Wu P. Li G. Yang W. Gutiérrez-Chico J. L. Tu S. (2023). Advances in diagnosis, therapy, and prognosis of coronary artery disease powered by deep learning algorithms. JACC Asia 3, 1–14. 10.1016/j.jacasi.2022.12.005 36873752 PMC9982227

[B20] De Siqueira F. R. Schwartz W. R. Pedrini H. (2013). Multi-scale gray level Co-occurrence matrices for texture description. Neurocomputing 120, 336–345. 10.1016/j.neucom.2012.09.042

[B21] Deng L. Platt J. (2014). “Ensemble deep learning for speech recognition,” in Proc. Interspeech, Singapore, 14-18 September 2014.

[B22] Dewangan N. K. Shukla S. (2015). A survey on ECG signal feature extraction and analysis techniques. Int. J. Innovative Res. Electr. Electron. Instrum. Control Eng. 3, 12–19.

[B23] Dinakarrao S. M. P. Jantsch A. Shafique M. (2019). Computer-aided arrhythmia diagnosis with bio-signal processing: A survey of trends and techniques. ACM Comput. Surv. (CSUR) 52, 1–37. 10.1145/3297711

[B24] Ebrahimi Z. Loni M. Daneshtalab M. Gharehbaghi A. (2020). A review on Deep Learning methods for ECG arrhythmia classification. Expert Syst. Appl. X 7, 100033. 10.1016/j.eswax.2020.100033

[B25] Esteban C. Staeck O. Baier S. Yang Y. Tresp V. (2016). “Predicting clinical events by combining static and dynamic information using recurrent neural networks,” in 2016 IEEE International Conference on Healthcare Informatics (ICHI) (Ieee), Chicago, 04-07 October 2016, 93–101.

[B26] Faust O. Shenfield A. Kareem M. San T. R. Fujita H. Acharya U. R. (2018). Automated detection of atrial fibrillation using long short-term memory network with RR interval signals. Comput. Biol. Med. 102, 327–335. 10.1016/j.compbiomed.2018.07.001 30031535

[B27] Flores N. Avitia R. L. Reyna M. A. García C. (2018). Readily available ECG databases. J. Electrocardiol. 51, 1095–1097. 10.1016/j.jelectrocard.2018.09.012 30497737

[B28] Fu L. Lu B. Nie B. Peng Z. Liu H. Pi X. (2020). Hybrid network with attention mechanism for detection and location of myocardial infarction based on 12-lead electrocardiogram signals. Sensors 20, 1020. 10.3390/s20041020 32074979 PMC7071130

[B29] Gabrié M. Manoel A. Luneau C. Macris N. Krzakala F. Zdeborová L. (2018). Entropy and mutual information in models of deep neural networks. Adv. Neural Inf. Process. Syst. 31.

[B30] Gao Z. Dang W. Wang X. Hong X. Hou L. Ma K. (2021). Complex networks and Deep Learning for EEG signal analysis. Cogn. Neurodynamics 15, 369–388. 10.1007/s11571-020-09626-1 PMC8131466 34040666

[B31] Haleem M. S. Castaldo R. Pagliara S. M. Petretta M. Salvatore M. Franzese M. (2021). Time adaptive ecg driven cardiovascular disease detector. Biomed. Signal Process. Control 70, 102968. 10.1016/j.bspc.2021.102968

[B32] Halford J. J. (2009). Computerized epileptiform transient detection in the scalp electroencephalogram: obstacles to progress and the example of computerized ECG interpretation. Clin. Neurophysiol. 120, 1909–1915. 10.1016/j.clinph.2009.08.007 19836303

[B33] Hammad M. Iliyasu A. M. Subasi A. Ho E. S. Abd El-Latif A. A. (2020). A multitier Deep Learning model for arrhythmia detection. IEEE Trans. Instrum. Meas. 70, 1–9. 10.1109/tim.2020.3033072 33776080

[B34] Hammad M. Kandala R. N. Abdelatey A. Abdar M. Zomorodi-Moghadam M. San Tan R. (2021). Automated detection of shockable ECG signals: A review. Inf. Sci. 571, 580–604. 10.1016/j.ins.2021.05.035

[B35] Han K. Wang Y. Chen H. Chen X. Guo J. Liu Z. (2022). A survey on vision transformer. IEEE Trans. Pattern Analysis Mach. Intell. 45, 87–110. 10.1109/TPAMI.2022.3152247 35180075

[B36] Hanbay K. (2019). Deep neural network based approach for ECG classification using hybrid differential features and active learning. IET Signal Process. 13, 165–175. 10.1049/iet-spr.2018.5103

[B37] Hausen R. Robertson B. E. (2020). Morpheus: A deep learning framework for the pixel-level analysis of astronomical image data. Astrophysical J. Suppl. Ser. 248, 20. 10.3847/1538-4365/ab8868

[B38] He Z. Yuan S. Zhao J. Du B. Yuan Z. Alhudhaif A. (2022). A novel myocardial infarction localization method using multi-branch densenet and spatial matching-based active semi-supervised learning. Inf. Sci. 606, 649–668. 10.1016/j.ins.2022.05.070

[B39] Hong J. Li H.-J. Yang C.-c. Han C.-L. Hsieh J.-c. (2022). A clinical study on atrial fibrillation, premature ventricular contraction, and premature atrial contraction screening based on an ECG deep learning model. Appl. Soft Comput. 126, 109213. 10.1016/j.asoc.2022.109213

[B40] Hong S. Zhou Y. Shang J. Xiao C. Sun J. (2020). Opportunities and challenges of deep learning methods for electrocardiogram data: A systematic review. Comput. Biol. Med. 122, 103801. 10.1016/j.compbiomed.2020.103801 32658725

[B41] Hu R. Chen J. Zhou L. (2022). A transformer-based deep neural network for arrhythmia detection using continuous ECG signals. Comput. Biol. Med. 144, 105325. 10.1016/j.compbiomed.2022.105325 35227968

[B42] Islam M. S. Islam M. N. Hashim N. Rashid M. Bari B. S. Al Farid F. (2022). New hybrid deep learning approach using BiGRU-BiLSTM and multilayered dilated CNN to detect arrhythmia. IEEE Access 10, 58081–58096. 10.1109/access.2022.3178710

[B43] Jiang M. Gu J. Li Y. Wei B. Zhang J. Wang Z. (2021). Hadln: hybrid attention-based deep learning network for automated arrhythmia classification. Front. Physiology 12, 683025. 10.3389/fphys.2021.683025 PMC8289344 34290619

[B44] Jun T. J. Nguyen H. M. Kang D. Kim D. Kim D. Kim Y.-H. (2018). ECG arrhythmia classification using a 2-D Convolutional Neural Network. *arXiv preprint arXiv:1804.06812* .

[B45] Khan M. A. Kim Y. (2021). Cardiac arrhythmia disease classification using lstm deep learning approach. Comput. Mater. Continua 67, 427–443. 10.32604/cmc.2021.014682

[B46] Kim Y. K. Lee M. Song H. S. Lee S.-W. (2022). Lessons from tough cases. IEEE Trans. Instrum. Meas. 71, 1–2. 10.5125/jkaoms.2022.48.1.1

[B47] Koppad D. (2021). Arrhythmia classification using deep learning: A review. WSEAS Trans. Biol. Biomed. 18, 96–105. 10.37394/23208.2021.18.11

[B48] Kumar S. Mallik A. Kumar A. Del Ser J. Yang G. (2023). Fuzz-ClustNet: coupled fuzzy clustering and deep neural networks for arrhythmia detection from ECG signals. Comput. Biol. Med. 153, 106511. 10.1016/j.compbiomed.2022.106511 36608461

[B49] Li Q. Rajagopalan C. Clifford G. D. (2013). Ventricular fibrillation and tachycardia classification using a machine learning approach. IEEE Trans. Biomed. Eng. 61, 1607–1613. 10.1109/TBME.2013.2275000 23899591

[B50] Li Y. Qian R. Li K. (2022). Inter-patient arrhythmia classification with improved deep residual convolutional neural network. Comput. Methods Programs Biomed. 214, 106582. 10.1016/j.cmpb.2021.106582 34933228

[B51] Liu F. Liu C. Zhao L. Zhang X. Wu X. Xu X. (2018). An open access database for evaluating the algorithms of electrocardiogram rhythm and morphology abnormality detection. J. Med. Imaging Health Inf. 8, 1368–1373. 10.1166/jmihi.2018.2442

[B52] Llamedo M. Martínez J. P. (2010). Heartbeat classification using feature selection driven by database generalization criteria. IEEE Trans. Biomed. Eng. 58, 616–625. 10.1109/TBME.2010.2068048 20729162

[B53] Lu Y. Jiang M. Wei L. Zhang J. Wang Z. Wei B. (2021). Automated arrhythmia classification using depthwise separable convolutional neural network with focal loss. Biomed. Signal Process. Control 69, 102843. 10.1016/j.bspc.2021.102843

[B54] Lui H. W. Chow K. L. (2018). Multiclass classification of myocardial infarction with convolutional and Recurrent Neural Networks for portable ECG devices. Inf. Med. Unlocked 13, 26–33. 10.1016/j.imu.2018.08.002

[B55] Luo K. Li J. Wang Z. Cuschieri A. (2017). Patient-specific deep architectural model for ECG classification. J. Healthc. Eng. 2017, 4108720. 10.1155/2017/4108720 29065597 PMC5499251

[B56] Luz E. J. d. S. Schwartz W. R. Cámara-Chávez G. Menotti D. (2016). ECG-based heartbeat classification for arrhythmia detection: A survey. Comput. Methods Programs Biomed. 127, 144–164. 10.1016/j.cmpb.2015.12.008 26775139

[B57] Ma S. Cui J. Xiao W. Liu L. (2022). Deep learning-based data augmentation and model fusion for automatic arrhythmia identification and classification algorithms. Comput. Intell. Neurosci. 2022, 1577778. 10.1155/2022/1577778 35990162 PMC9388256

[B58] Mahmud T. Fattah S. A. Saquib M. (2020). Deeparrnet: an efficient deep CNN architecture for automatic arrhythmia detection and classification from denoised ECG beats. IEEE Access 8, 104788–104800. 10.1109/access.2020.2998788

[B59] Majumdar A. Ward R. (2017). “Robust greedy deep dictionary learning for ECG arrhythmia classification,” in 2017 International Joint Conference on Neural Networks (IJCNN) (IEEE), Anchorage, 14-19 May 2017.

[B60] Mar T. Zaunseder S. Martínez J. P. Llamedo M. Poll R. (2011). Optimization of ECG classification by means of feature selection. IEEE Trans. Biomed. Eng. 58, 2168–2177. 10.1109/TBME.2011.2113395 21317067

[B61] Mathur P. Srivastava S. Xu X. Mehta J. L. (2020). Artificial intelligence, machine learning, and cardiovascular disease. Clin. Med. Insights Cardiol. 14, 1179546820927404. 10.1177/1179546820927404 32952403 PMC7485162

[B62] Midani W. Ouarda W. Ayed M. B. (2023). DeepArr: an investigative tool for arrhythmia detection using a contextual deep neural network from electrocardiograms (ECG) signals. Biomed. Signal Process. Control 85, 104954. 10.1016/j.bspc.2023.104954

[B63] Montesinos-López O. A. Montesinos-López A. Pérez-Rodríguez P. Barrón-López J. A. Martini J. W. Fajardo-Flores S. B. (2021). A review of deep learning applications for genomic selection. BMC Genomics 22, 19–23. 10.1186/s12864-020-07319-x 33407114 PMC7789712

[B64] Moody G. B. Mark R. G. (1990). “The mit-bih arrhythmia database on cd-rom and software for use with it,” in 1990 Proceedings Computers in Cardiology (IEEE), Chicago, 23-26 September 1990.

[B65] Moody G. Mark R. (2001). The impact of the mit-bih arrhythmia database. IEEE Eng. Med. Biol. Mag. 20, 45–50. 10.1109/51.932724 11446209

[B66] Moody G. Muldrow W. Mark R. (1984). The mit-bih noise stress test database. Comput. Cardiol., 381–384.

[B67] Murat F. Yildirim O. Talo M. Baloglu U. B. Demir Y. Acharya U. R. (2020). Application of Deep Learning techniques for heartbeats detection using ECG signals-analysis and review. Comput. Biol. Med. 120, 103726. 10.1016/j.compbiomed.2020.103726 32421643

[B68] Nayan N. A. Ab Hamid H. (2019). Evaluation of patient electrocardiogram datasets using signal quality indexing. Bull. Electr. Eng. Inf. 8, 519–526. 10.11591/eei.v8i2.1289

[B69] Nguyen M. T. Nguyen B. V. Kim K. (2018). Deep feature learning for sudden Cardiac Arrest detection in automated external defibrillators. Sci. Rep. 8, 17196–17212. 10.1038/s41598-018-33424-9 30464177 PMC6249221

[B70] Nurmaini S. Darmawahyuni A. Sakti Mukti A. N. Rachmatullah M. N. Firdaus F. Tutuko B. (2020a). Deep learning-based stacked denoising and autoencoder for ecg heartbeat classification. Electronics 9, 135. 10.3390/electronics9010135

[B71] Nurmaini S. Tondas A. E. Darmawahyuni A. Rachmatullah M. N. Partan R. U. Firdaus F. (2020b). Robust detection of atrial fibrillation from short-term electrocardiogram using convolutional neural networks. Future Gener. Comput. Syst. 113, 304–317. 10.1016/j.future.2020.07.021

[B72] Oh S. Lee M. (2022). A shallow domain knowledge injection (sdk-injection) method for improving cnn-based ecg pattern classification. Appl. Sci. 12, 1307. 10.3390/app12031307

[B73] Ojha M. K. Wadhwani S. Wadhwani A. K. Shukla A. (2022). Automatic detection of arrhythmias from an ECG signal using an auto-encoder and SVM classifier. Phys. Eng. Sci. Med. 45, 665–674. 10.1007/s13246-022-01119-1 35304901

[B74] Özbay Y. Ceylan R. Karlik B. (2006). A fuzzy clustering neural network architecture for classification of ecg arrhythmias. Comput. Biol. Med. 36, 376–388. 10.1016/j.compbiomed.2005.01.006 15878480

[B75] Parvaneh S. Rubin J. Babaeizadeh S. Xu-Wilson M. (2019). Cardiac arrhythmia detection using deep learning: A review. J. Electrocardiol. 57, S70-S74–S74. 10.1016/j.jelectrocard.2019.08.004 31416598

[B76] Peimankar A. Puthusserypady S. (2021). DENS-ECG: A deep learning approach for ECG signal delineation. Expert Syst. Appl. 165, 113911. 10.1016/j.eswa.2020.113911

[B77] Penzel T. Moody G. B. Mark R. G. Goldberger A. L. Peter J. H. (2000). “The apnea-ecg database,” in Computers in Cardiology 2000, Cambridge, 24-27 September 2000.

[B78] Pudil P. Novovičová J. Kittler J. (1994). Floating search methods in feature selection. Pattern Recognit. Lett. 15, 1119–1125. 10.1016/0167-8655(94)90127-9

[B79] Sannino G. De Pietro G. (2018). A Deep Learning approach for ECG-based heartbeat classification for arrhythmia detection. Future Gener. Comput. Syst. 86, 446–455. 10.1016/j.future.2018.03.057

[B80] Sarikaya R. Hinton G. E. Deoras A. (2014). Application of deep belief networks for natural language understanding. IEEE/ACM Trans. Audio, Speech, Lang. Process. 22, 778–784. 10.1109/taslp.2014.2303296

[B81] Sepahvand M. Abdali-Mohammadi F. (2022). A novel method for reducing arrhythmia classification from 12-lead ECG signals to single-lead ECG with minimal loss of accuracy through teacher-student knowledge distillation. Inf. Sci. 593, 64–77. 10.1016/j.ins.2022.01.030

[B82] Sharma P. Dinkar S. K. Gupta D. (2021). A novel hybrid Deep Learning method with cuckoo search algorithm for classification of arrhythmia disease using ECG signals. Neural Comput. Appl. 33, 13123–13143. 10.1007/s00521-021-06005-7

[B83] Sujadevi V. Soman K. Vinayakumar R. (2017). “Detection of Atrial Fibrillation from short time single lead ECG traces using Recurrent Neural Networks,” in Intelligent systems technologies and applications (Germany: Springer), 212–221.

[B84] Sun J.-Y. Shen H. Qu Q. Sun W. Kong X.-Q. (2021). The application of Deep Learning in electrocardiogram: where we came from and where we should go? Int. J. Cardiol. 337, 71–78. 10.1016/j.ijcard.2021.05.017 34000355

[B85] Taddei A. Distante G. Emdin M. Pisani P. Moody G. Zeelenberg C. (1992). The european st-t database: standard for evaluating systems for the analysis of st-t changes in ambulatory electrocardiography. Eur. Heart J. 13, 1164–1172. 10.1093/oxfordjournals.eurheartj.a060332 1396824

[B86] Taji B. Chan A. D. Shirmohammadi S. (2017). False alarm reduction in atrial fibrillation detection using deep belief networks. IEEE Trans. Instrum. Meas. 67, 1124–1131. 10.1109/tim.2017.2769198

[B87] Tan J. H. Hagiwara Y. Pang W. Lim I. Oh S. L. Adam M. (2018). Application of stacked convolutional and long short-term memory network for accurate identification of cad ecg signals. Comput. Biol. Med. 94, 19–26. 10.1016/j.compbiomed.2017.12.023 29358103

[B88] Teich M. C. Lowen S. B. Jost B. M. Vibe-Rheymer K. Heneghan C. (2000). Heart rate variability: Measures and models. New Jersey: John Wiley Sons, Ltd. chap. 6. 159–213. 10.1109/9780470545379.ch6

[B89] Teplitzky B. A. McRoberts M. Ghanbari H. (2020). Deep Learning for comprehensive ECG annotation. Heart rhythm. 17, 881–888. 10.1016/j.hrthm.2020.02.015 32354454 PMC9247885

[B90] Tihonenko V. Khaustov A. Ivanov S. Rivin A. Yakushenko E. (2008). St petersburg incart 12-lead arrhythmia database. PhysioBank PhysioToolkit PhysioNet.

[B91] Tolstikhin I. O. Houlsby N. Kolesnikov A. Beyer L. Zhai X. Unterthiner T. (2021). Mlp-mixer: an all-mlp architecture for vision. Adv. Neural Inf. Process. Syst. 34, 24261–24272.

[B92] Torralba A. Efros A. A. (2011). “Unbiased look at dataset bias,” in CVPR 2011, Colorado, 20-25 June 2011.

[B93] Ullah A. Anwar S. M. Bilal M. Mehmood R. M. (2020). Classification of arrhythmia by using Deep Learning with 2-D ECG spectral image representation. Remote Sens. 12, 1685. 10.3390/rs12101685

[B94] Wang G. Zhang C. Liu Y. Yang H. Fu D. Wang H. (2019). A global and updatable ECG beat classification system based on Recurrent Neural Networks and Active Learning. Inf. Sci. 501, 523–542. 10.1016/j.ins.2018.06.062

[B95] Wang L. Zhou X. (2019). Detection of congestive heart failure based on LSTM-based deep network via short-term RR intervals. Sensors 19, 1502. 10.3390/s19071502 30925693 PMC6480269

[B96] Wen X. Huang Y. Wu X. Zhang B. (2019). “A correlation-based algorithm for beat-to-beat heart rate estimation from ballistocardiograms,” in 2019 41st Annual International Conference of the IEEE Engineering in Medicine and Biology Society, Germany, 23-27 July 2019. 10.1109/EMBC.2019.8856464 31947296

[B97] Wu Q. Liu Y. Li Q. Jin S. Li F. (2017). The application of Deep Learning in computer vision. In 2017 Chinese Automation Congress (CAC), China, 20-22 October 2017.

[B98] Xia Y. Wulan N. Wang K. Zhang H. (2017). “Atrial Fibrillation detection using stationary wavelet transform and Deep Learning,” in 2017 Computing in Cardiology (CinC), France, 24-27 September 2017.

[B99] Xia Y. Xie Y. (2019). A novel wearable electrocardiogram classification system using Convolutional Neural Networks and active learning. IEEE Access 7, 7989–8001. 10.1109/access.2019.2890865

[B100] Xia Y. Zhang H. Xu L. Gao Z. Zhang H. Liu H. (2018). An automatic cardiac arrhythmia classification system with wearable electrocardiogram. IEEE Access 6, 16529–16538. 10.1109/access.2018.2807700

[B101] Xiang Y. Lin Z. Meng J. (2018). Automatic QRS complex detection using two-level Convolutional Neural Network. Biomed. Eng. Online 17, 13–17. 10.1186/s12938-018-0441-4 29378580 PMC5789562

[B102] Xiao Q. Lee K. Mokhtar S. A. Ismail I. Pauzi A. L. b. M. Zhang Q. (2023a). Deep learning-based ECG arrhythmia classification: A systematic review. Appl. Sci. 13, 4964. 10.3390/app13084964

[B103] Xiao Q. Lee K. Mokhtar S. A. Ismail I. Pauzi A. L. b. M. Zhang Q. (2023b). Deep learning-based ecg arrhythmia classification: A systematic review. Appl. Sci. 13, 4964. 10.3390/app13084964

[B104] Yan G. Liang S. Zhang Y. Liu F. (2019). “Fusing transformer model with temporal features for ECG heartbeat classification,” in 2019 IEEE International Conference on Bioinformatics and Biomedicine, San Diego, 18-21 November 2019.

[B105] Yildirim Ö. (2018). A novel wavelet sequence based on deep bidirectional LSTM network model for ECG signal classification. Comput. Biol. Med. 96, 189–202. 10.1016/j.compbiomed.2018.03.016 29614430

[B106] Zahid M. U. Kiranyaz S. Gabbouj M. (2022). Global ECG classification by self-operational neural Networks with feature injection. IEEE Trans. Biomed. Eng. 70, 205–215. 10.1109/TBME.2022.3187874 35786545

[B107] Zhang C. Wang G. Zhao J. Gao P. Lin J. Yang H. (2017). “Patient-specific ECG classification based on recurrent neural networks and clustering technique,” in 2017 13th IASTED International Conference on Biomedical Engineering, Austria, 20-21 February 2017.

[B108] Zhang H. Liu C. Tang F. Li M. Zhang D. Xia L. (2023). Atrial fibrillation classification based on the 2d representation of minimal subset ecg and a non-deep neural network. Front. Physiology 14, 182. 10.3389/fphys.2023.1070621 PMC9971936 36866172

[B109] Zhang Z. Dong J. Luo X. Choi K.-S. Wu X. (2014). Heartbeat classification using disease-specific feature selection. Comput. Biol. Med. 46, 79–89. 10.1016/j.compbiomed.2013.11.019 24529208

[B110] Zhao Z. Murphy D. Gifford H. Williams S. Darlington A. Relton S. D. (2022). Analysis of an adaptive lead weighted resnet for multiclass classification of 12-lead ecgs. Physiol. Meas. 43, 034001. 10.1088/1361-6579/ac5b4a 35255483

[B111] Zhou X. Zhu X. Nakamura K. Mahito N. (2018). “Premature ventricular contraction detection from ambulatory ECG using recurrent neural networks,” in 2018 40th Annual International Conference of the IEEE Engineering in Medicine and Biology Society, Honolulu, 18-21 July 2018. 10.1109/EMBC.2018.8512858 30440928

[B112] Zhu J. Lv J. Kong D. (2022). Cnn-fws: A model for the diagnosis of normal and abnormal ecg with feature adaptive. Entropy 24, 471. 10.3390/e24040471 35455133 PMC9025839

